# Genetic control of rhizosphere microbiome of the cotton plants under field conditions

**DOI:** 10.1007/s00253-024-13143-0

**Published:** 2024-06-11

**Authors:** Feng Wei, Zili Feng, Chuanzhen Yang, Lihong Zhao, Yalin Zhang, Jinglong Zhou, Hongjie Feng, Heqin Zhu, Xiangming Xu

**Affiliations:** 1 National Key Laboratory of Cotton Bio-Breeding and Integrated Utilization, Institute of Cotton Research of Chinese Academy of Agricultural Sciences, Anyang, 455000 China; 2 Western Agricultural Research Center, Chinese Academy of Agricultural Sciences, Changji, 831100 Xinjiang China; 3 School of Agricultural Sciences, Zhengzhou University, Zhengzhou, 450001 China; 4 NIAB East Malling Research, Kent, ME19 6BJ UK

**Keywords:** Cotton, Microbiome, Rhizosphere soil, Heritability, Verticillium wilt

## Abstract

**Abstract:**

Understanding the extent of heritability of a plant-associated microbiome (phytobiome) is critically important for exploitation of phytobiomes in agriculture. Two crosses were made between pairs of cotton cultivars (Z2 and J11, L1 and Z49) with differential resistance to Verticillium wilt. F_2_ plants were grown in a field, together with the four parents to study the heritability of cotton rhizosphere microbiome. Amplicon sequencing was used to profile bacterial and fungal communities in the rhizosphere. F_2_ offspring plants of both crosses had higher average alpha diversity indices than the two parents; parents differed significantly from F_2_ offspring in Bray–Curtis beta diversity indices as well. Two types of data were used to study the heritability of rhizosphere microbiome: principal components (PCs) and individual top microbial operational taxonomic units (OTUs). For the L1 × Z49 cross, the variance among the F_2_ progeny genotypes (namely, genetic variance, *V*_*T*_) was significantly greater than the random variability (*V*_*E*_) for 12 and 34 out of top 100 fungal and bacterial PCs, respectively. For the Z2 × J11 cross, the corresponding values were 10 and 20 PCs. For 29 fungal OTUs and 10 bacterial OTUs out of the most abundant 100 OTUs, genetic variance (*V*_*T*_) was significantly greater than *V*_*E*_ for the L1 × Z49 cross; the corresponding values for the Z2 × J11 cross were 24 and one. The estimated heritability was mostly in the range of 40% to 60%. These results suggested the existence of genetic control of polygenic nature for specific components of rhizosphere microbiome in cotton.

**Key points:**

• *F*_*2*_ *offspring cotton plants differed significantly from parents in rhizosphere microbial diversity.*

• *Specific rhizosphere components are likely to be genetically controlled by plants.*

• *Common PCs and specific microbial groups are significant genetic components between the two crosses.*

**Supplementary Information:**

The online version contains supplementary material available at 10.1007/s00253-024-13143-0.

## Introduction

The plant-associated microbiome (phytobiome) may benefit their host plants in several aspects (Goh et al. 2013), including preventing pathogens and pests (Chapelle et al. 2016; Mendes et al. 2013; Wei et al. 2021, 2019), enhancing tolerance to drought and nutrient stress (Coleman-Derr and Tringe 2014), changing flowering time (Lu et al. 2018), and improving plant productivity (Berendsen et al. 2012). The microbiome structure in the rhizosphere often differs across plant species (Naylor et al. 2017), as well as among genotypes within a single species (Wei et al. 2019). Recent studies have shown that rhizosphere microbiomes are shaped, to a certain extent, by host genetics (Edwards et al. 2015; Peiffer et al. 2013; Schlaeppi et al. 2014). A study comparing the root microbiomes of cereal crops showed that there is a strong correlation between host genetic differences and microbiome composition, indicating that a subset of phytobiomes may be affected by host genotypes across a series of plant hosts (Naylor et al. 2017). Microbiomes can function as a phenotypically plastic buffer between host genotype and environment, and interact with environment to shape host phenotypes (Mueller and Sachs 2015). Therefore, expression of almost any host phenotype depends to some extent on phytobiome composition (Mueller and Sachs 2015). Although, there is consistent evidence of interactions between host genotype and phytobiome composition, identifying specific genetic factors driving/controlling phytobiome acquisition and assembly remains a challenge.

The main focus of plant breeding has been on the improvement of crop productivity, quality and pest/disease resistance; modern breeding has been shown to reduce genetic diversity of modern crops (Busby et al. 2017; Pérez-Jaramillo et al. 2016). This may have unintended consequences on the phytobiome associated with the reduced crop diversities. The importance of the rhizosphere microbiome in the plant ecosystem functioning has been widely recognized, but plants have been bred by altering their genomic information with rarely consideration of their interaction with surrounding organisms. Recently, there has been a paradigm shift in considering plants as a holobiont, an ecological and evolutionary unit containing both the host and its microbiome (Wei and Jousset 2017). Thus, we may need to include the ability of recruiting and interacting with beneficial microbes through root exudates as a selection criterion (Kroll et al. 2017; Wille et al. 2019). Mendes et al. (2018) have shown that resistance breeding in common bean has unintentionally co-selected for plant traits that strengthen the rhizosphere microbiome network structure and enrich for specific beneficial bacterial genera that express antifungal traits involved in plant protection against infections by soilborne pathogens. Identifying these plant traits and microbial taxonomies will help breeders to select for plant traits that enrich desired microbial groups (Mendes et al. 2018). However, to pursue this route, we also need to understand the magnitude of heritability of microbiome and the nature of such genetic control.

Heritable components of microbiome variation may reflect plant–microbe interactions, which are formed through natural selection acting on plant traits underlying fitness. Recently, Wagner et al. (2020) found that inbred lines and hybrids differ consistently in composition of bacterial and fungal rhizosphere communities of maize. Most studies on microbiome composition in complex environments have observed that the interactions between genotype and environment are at least as strong as the main genotypic effects (Lundberg et al. 2016; Peiffer et al. 2013; Wagner et al. 2020; Walters et al. 2018). Currently, most studies on host genotype effects on microbiome have shown that there are differences in microbiomes composition among varieties or accessions with no well-defined genetic relationship to each other, often chosen to represent a breadth of diversity within the host species (Clouse and Wagner 2021). Thus, there is a lack of purposely designed studies to investigate heritability of phytobiomes through designed crosses between plant genotypes. The information on microbiome heritability obtained from specific crosses may inform breeders about potential selection gains that can be achieved in breeding programs.

Cotton Verticillium wilt, caused by *Verticillium dahliae*, is one of the devastating plant diseases worldwide. Because of the inaccessibility of *V*. *dahliae* during infection, long-term survival of its microsclerotia in soil, its broad host range and withdrawal of broad-spectrum soil fumigants, it is difficult to control Verticillium wilt (Klosterman et al. 2009). Recently, we demonstrated that the genotypic response to *V*. *dahliae* in cotton is associated with many microbial groups in rhizosphere and endophytic microbiomes (Wei et al. 2019), suggesting the potential of improved phytobiomes through breeding for wilt disease resistance.

In the present study, we selected two pairs of cotton cultivars with differential resistance to *V. dahliae* as parents [L1 (susceptible) × Z49 (resistant) and Z2 (resistant) × J11 (susceptible)] to determine to what extend the rhizosphere microbiome is heritable and hence amendable to selection in breeding programs. We raised and have grown 100 F_2_ offspring plants from each cross, together with the four parental plants in a field site, and used amplicon sequencing to profile bacterial and fungal communities in the rhizosphere.

## Materials and methods

### Plant materials

**Fig. 1:**
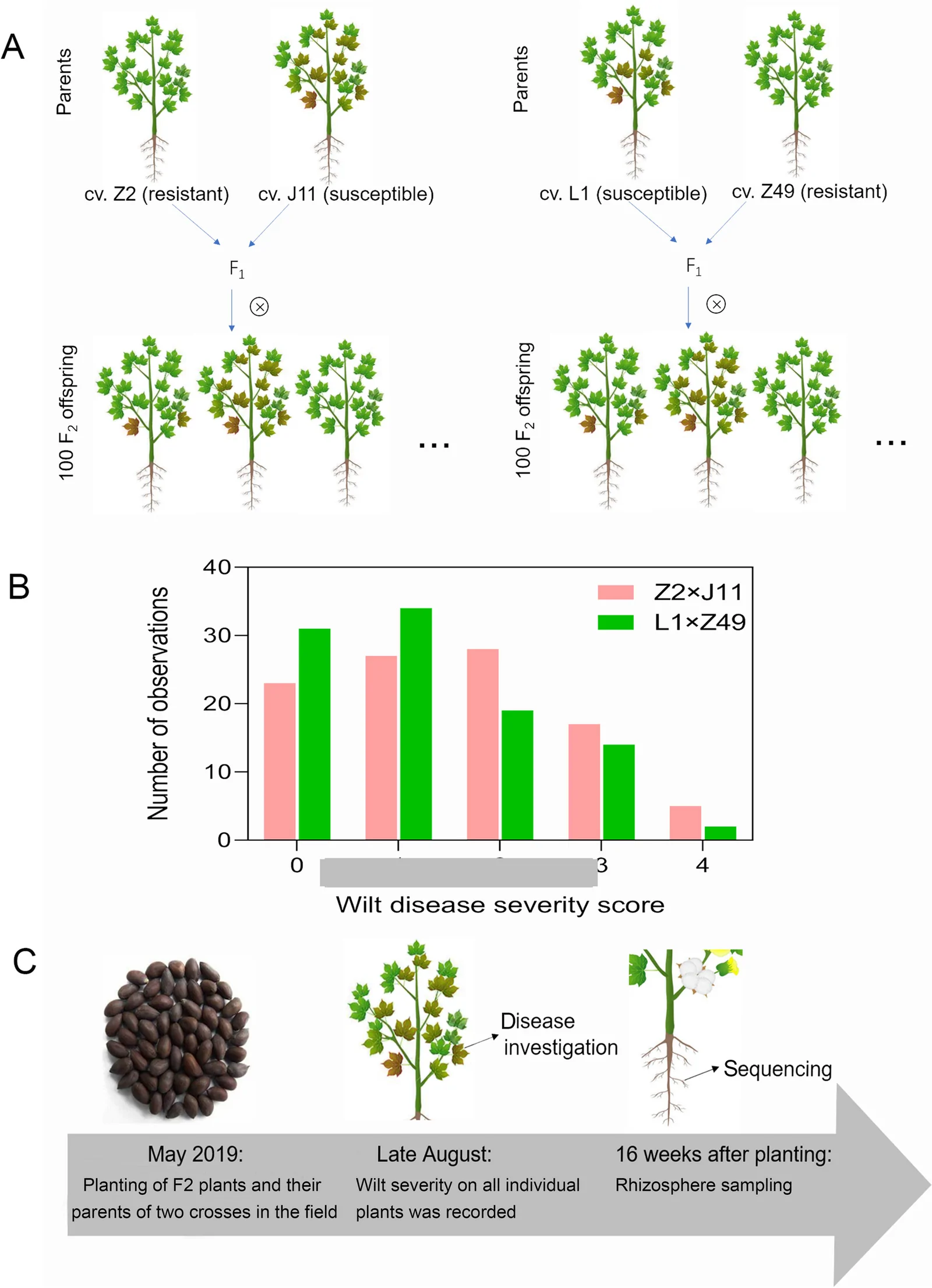
Overview of the experimental approach. **A** Two crosses were made between pairs of cotton cultivars with differential resistance to Verticillium wilt. F_2_ plants were grown together with the four parents in field trials; **B** wilt severity scores of all 100 individual F_2_ progeny plants for the each cross, recorded on the 22th of August 2019; and **C** timelines of key experimental tasks

Cotton (*Gossypium hirsutum*) seeds were obtained from Mid-term Gene Bank in Cotton Research Institute of the Chinese Academy of Agricultural Sciences, Anyang, China. Two resistant and two susceptible cotton cultivars were chosen as parents for crossing: Zhongzhimain2 (Z2, resistant against *V. dahliae*) crossed with Jimian11 (J11, susceptible) and LocalVIR875-1 (L1, susceptible) with Zhongmiansuo49 (Z49, resistant) (Fig. 1A). The parent materials used in this study were both continuously self-pollinated for 6 generations. In order to ensure the purity of F_1_ seeds, the hybridization process was carried out strictly through a procedure of emasculation on the afternoon of the first day and pollination on the morning of the second day. F_2_ seeds were obtained from F_1_ hybrids.

On 28 April 2018, the four cotton cultivars were planted in a field (36°03′44″ N, 114°28′52″ E) at the Institute of Cotton Research, Chinese Academy of Agricultural Sciences (Anyang, China). The field, used for evaluating cotton cultivar resistance against *V. dahliae*, was artificially inoculated with *V. dahliae* 20 years ago. The soil at the site is classified as cambisol type soil. The two crosses (Z2 × J11 and L1 × 49) were made and the F_1_ hybrids were sown in a field (18°17′10″ N, 109°29′52″ E) in Hainan Province, China on the 9th of November 2018 to generate F_2_ seeds for investigation of the heritability of rhizosphere microbiome.

### Site description and field experiment design

On the 30th of April 2019, a field experiment was set up at the Institute of Cotton Research, Chinese Academy of Agricultural Sciences (Anyang, China) (Fig. 1C). A completely randomized block design with four blocks was used. Within each block, there were six plots of 5 m in length with two rows (inter-row distance of 0.8 m); neighboring plots were separated by 0.8 m. With each block, individual plots were randomly assigned to the six groups of plants: four parents and two F_2_ populations. There were 25 plants within each plot; for each cross, 25 of the 100 F_2_ plants were randomly allocated to each of the four blocks.

On the 22th of August 2019, wilt severity of all individual plants was recorded on a scale of 0 to 4 as described previously (Wei et al. 2019). For cv. Z2, there were 68, 19, 11, 2, and 0 plants with severity score of 0, 1, 2, 3, and 4, respectively; the corresponding values for cv. J11 were 8, 8, 22, 51, and 11 plants; for cv. L1 were 15, 13, 25, 39, and 8 plants; and for cv. Z49 were 52, 29, 15, 4, and 0 plants (Supplemental Table S1). For Z2 × J11 cross, there were 23, 27, 28, 17, and 5 plants with severity score of 0, 1, 2, 3, and 4, respectively; the corresponding values for L1 × Z49 cross were 31, 34, 19, 14, and 2 plants (Fig. 1B; Supplemental Table S1).

### Rhizosphere sample collection

On the 24th of August 2019 (at the boll-forming stage), approximately 16 weeks after sowing (Fig. 1C), rhizosphere soil samples were collected and stored as described previously (Wei et al. 2019). For F_2_, all plants were sampled (i.e., 100 plants for each cross); for each of the four parents, only four plants were sampled from each block, giving 16 plants per parental genotype. Fewer samples were obtained for the four parents since they are genetically (or very close) homozygous; the parental samples were thus used to estimate environmental variability (i.e., variability due to all non-host-genotypic effects). Because individual F_2_ plants differed in their genotypes, variability among F_2_ plants also included the genetic origin in addition to the environmental origin as for the parents.

Each plant was removed from the soil with a spade. Root systems of the plants were first vigorously shaken to remove loosely adhering soil particles. Plant fine roots were cut into pieces of approximately 2-cm length with a pair of sterile scissors. Rhizosphere soil samples were harvested in 500-ml screw-cap bottles with ca. 20 g roots. Each bottle was filled up to 300 ml with 1:50 TE buffer (1 M Tris, 500 mM EDTA, and 1.2% Triton diluted in sterile distilled water) and shaken at 270 rpm for 1 h. The root-washing suspension was filtered with sterile cheesecloth and centrifuged at 4000 × *g* for 20 min (Wei et al. 2019). The supernatant was discarded by pipetting. This procedure was repeated three times before the pellets were re-suspended in the remaining solution, transferred to a 2-ml Eppendorf tube and centrifuged at 14,000 × *g* for 20 min. The pellets were immediately frozen and stored at − 80 °C before DNA extraction.

### DNA extraction and sequencing

Soil pellets were re-suspended in 500 μl MoBio PowerSoil bead solution, and DNA was extracted from the resulting pellets (250 mg) using the MoBio PowerSoil DNA Isolation Kit (MoBio Laboratories, Carlsbad, CA, USA) according to the manufacturer’s protocol. The extracts were checked on a 1% agarose gel; DNA concentration was estimated by a NanoDrop ND-2000 spectrophotometer (NanoDrop Technologies, Wilmington, DE, USA). DNA was stored at − 80 °C until further analysis.

For bacteria, the V3-V4 hypervariable region of the 16S rRNA gene was amplified in triplicates for each sample using the 341F/805R primers (Herlemann et al. 2011). For fungi, primers ITS5/ITS2 (White et al. 1990) were used to amplify the ITS1 region in triplicates for each sample. PCR reactions and the extraction and purification of amplicons followed previously established methods (Wei et al. 2019). Sequencing libraries were generated with the Ion Plus Fragment Library Kit 48 rxns (Thermo Scientific, Waltham, USA) following the manufacturer’s recommendations. The quality of each library was assessed on a Qubit 2.0 Fluorometer (Life Technologies, Carlsbad, California, USA). Finally, total DNA were submitted to Novogene Co. Ltd. (Beijing, China) for Illumina sequencing, the libraries were sequenced on an Ion S5™ XL platform (Thermo Fisher Scientific, Waltham, MA) to generate single-end reads. The resulting library consisted of 528 samples that were sequenced: 264 rhizosphere samples (2 crosses × 100 F_2_ plants + 4 parents × 16 replicates) each for 16S rRNA and fungal ITS sequences.

### Sequencing processing and taxonomy assignment

The clear reads from the service provider were used to generate operational taxonomic units (OTUs) and assign taxonomy, following an established pipeline (Wei et al. 2019). Briefly, high-quality sequences were obtained by quality control and filtering of sequence quality with very stringent criteria following our previous publication (Deakin et al. 2018), and this was carried out separately for the two type of data sets (16S rRNA and ITS). High-quality sequences were first dereplicated, and unique sequences with only one read were discarded. Then all unique sequence reads were sorted by their respective frequencies and then grouped into OTUs based on 97% or greater identity. All OTU were processed using the UPARSE pipeline (Version 10.0) (Edgar 2013) unless specified otherwise. The clustering algorithm also removed chimeras. The raw reads annotated as mitochondria or chloroplasts and were also removed. The SINTAX algorithm (https://www.drive5.com/usearch/manual/sintax_algo.html) then assigned each OTU representative sequence to taxonomic ranks by alignment with the gene sequences against the Unite V7 fungal database (Kõljalg et al. 2013) and the RDP training set (v16) bacterial database (Love et al. 2014). Then, an OTU table (a sample-by-observation contingency table) was generated by aligning all sequences filtered with far less stringent criteria with the OTU representative sequences as described by Deakin et al. (2018).

### Statistical data analysis

The median-of-ratios method implemented in DESeq2 (Anders and Huber 2010; Love et al. 2014) was used to normalize the OTU counts before any statistical analysis. All statistical analyses were carried out in R 4.0.3 (R Core Team 2021). There were six groups of samples, classified into two hierarchical levels: two crosses (Z2 × J11 and L1 × Z49), and three groups within each cross (two parents and F_2_ progeny).

Alpha (*α*) diversity (Shannon and Simpson) indices were calculated using the R vegan 2.3–1 package (Dixon 2003). The rank of α diversity indices were subjected to ANOVA to assess the differences between two crosses and between three groups within each cross via a permutation test for significance. Beta (*β*) diversity indices were calculated as Bray–Curtis indices and then subjected to ANOVA between parents and between progeny genotypes with a permutation test based on pseudo-*F* ratios (implemented as the Adonis procedure in the vegan package (Dixon 2003)).

To determine whether there are significant genotypic effects among the 100 F_2_ progeny plants from each cross, an *F* test was carried out to compare two variance estimates for the trait under investigation (e.g., counts for specific microbial OTUs): the variance [*V*_*T*_] among the 100 progeny samples, and the variance within the four parents. As explained above, *V*_*T*_ includes both origins of host genotypes and other non-host-genotype [*V*_*E*_] effects. *V*_*E*_ was estimated as the residual variance from the ANOVA of the four parents, each with 16 replicates; thus *V*_*E*_ estimate had 60 degrees of freedom. If the F test showed that *V*_*T*_ is significantly greater than *V*_*E*_, then the genetic variance [*V*_*G*_] component was estimated as (*V*_*T*_ − *V*_*E*_). Consequently, broad sense heritability was estimated as *V*_*G*_/*V*_*T*_.

Two datasets were used for analysis of genetic components: principal component (PC) scores and the normalized data of those OTUs with the highest counts (relative abundance). To generate PC scores, only those OTUs with the highest counts accounting for 99.99% of the total normalized counts were retained for PC analysis (PCA). Before PCA, the normalized counts data were first logarithm transformed on the natural base and then standardized. The 100 OTUs with the highest counts were subjected to genetic component analysis. For each PC or OTU, *V*_*E*_ was estimated from the parent samples and *V*_*T*_ from the 100 progeny samples. Similarly, two single degree contrasts were used to test whether the two parents of each cross differ significantly. Within the analysis of each data type (PC or OTU), the Benjamini-Hochberg (BH) adjustment was used to correct for the false discovery rate associated with the multiple testing. Statistical significance was determined at the 5% level (BH adjusted). BLASTn searches against the GenBank non-redundant database was then used to further characterize those OTUs with significant genetic components.

## Results

### Sequence quality and generation of OTUs

**Fig. 2:**
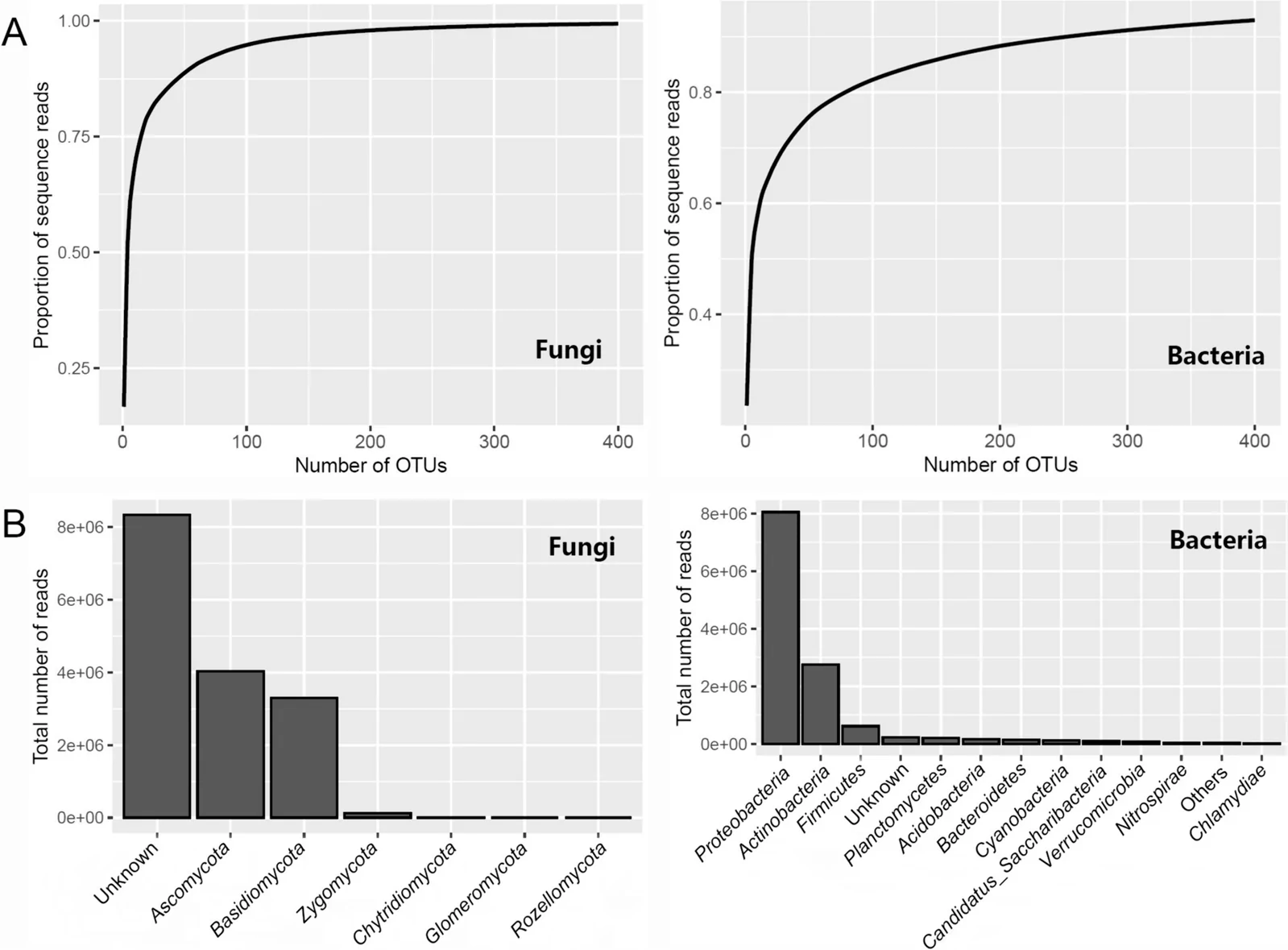
Overall sequencing results and taxonomic composition of bacterial and fungal rhizosphere microbiomes. **A** Proportion of the cumulative sequence reads plotted against the number of fungal OTUs and bacterial OTUs, where the OTUs were sorted in the descending order with respect to the number of their sequence reads. **B** Taxonomic composition of bacterial and fungal microbiomes. Histogram of the number of the ITS and 16S rRNA sequences that were assigned to the phylum at the 90% confidence level; the “Unknown” group consists of those OTUs that cannot be assigned to a unique phylum

For L1 × Z49 samples (including the two parents), the number of fungal raw reads ranged from 52,163 to 92,596 per sample, with an average of 80,795. Good quality reads accounted for > 96% of the raw reads, ranging from 49,956 to 89,182 per sample with an average of 77 718. For Z2 × J11 samples (including the two parents), the number of fungal raw reads ranged from 52,009 to 89,799 per sample, with an average of 78,051. Good quality reads accounted for > 96% of the raw reads, ranging from 49,603 to 86,987 per sample with an average of 75,119. Average read length was 221 and 222 bp for the L1 × Z49 and Z2 × J11 crosses, respectively. There were 1,487 fungal OTUs. Number of sequences classified into fungal OTUs ranged from 31,853 to 77,683 per sample, with an average of 59,781. Sequencing depth was sufficient for all samples (Supplemental Fig. S1). Although there were 1487 fungal OTUs, most fungal reads came from fewer than 100 OTUs. The most common OTU accounted for 16.7% of the total number of sequences; the top four and 51 fungal OTUs accounted for more than 50% and 90% of the total number of sequences, respectively (Fig. 2A). Nearly 53% of the fungal sequences could not be assigned to the phylum level at the 90% confidence level (Fig. 2B); most of the other sequences were assigned to *Ascomycota* (25.5%) or *Basidiomycota* (20.9%) (Fig. 2B).

For L1 × Z49 samples (including parents), the number of bacterial raw reads per sample ranged from 49,813 to 108,445 with an average of 71,309, of which more than 85% were of good quality reads, ranging from 39,759 to 88,548 per sample with an average of 60,298. For Z2 × J11 samples (including parents), the number of bacterial raw reads ranged from 48,575 to 100,987 per sample (average—76,404); good quality reads accounted for > 84% of the raw reads, ranging from 38,795 to 87,633 per sample (average—64,492). The average read length for both crosses was 418 bp. There were 4196 bacterial OTUs. The number of sequences classified into bacterial OTUs ranged from 27,189 to 77,162 per sample, with an average of 47,371. Sequencing depth was sufficient for all samples (Supplemental Fig. S1). The most common OTU accounted for 23.5% of the total sequences, with the top five and 252 bacterial OTUs accounting for more than 50% and 90% of the total number of sequences, respectively (Fig. 2A). The dominant bacterial phylum was *Proteobacteria*, accounting for 64.4% of the sequence reads, followed by *Actinobacteria* (22.0%) and *Firmicutes* (4.9%) (Fig. 2B).

### Alpha and beta diversity of microbial communities in rhizosphere

**Fig. 3:**
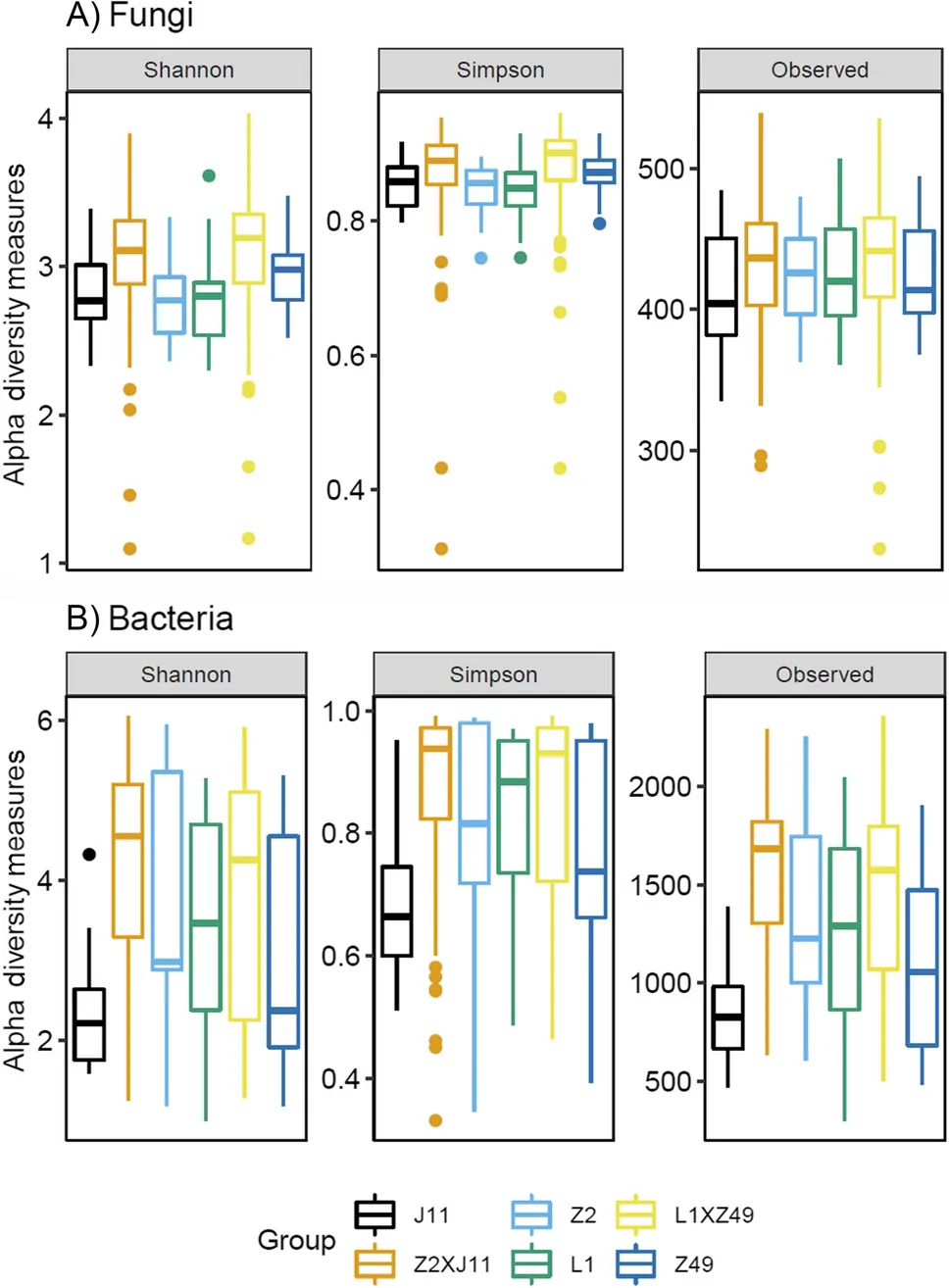
Alpha diversity measures of cotton rhizosphere microbiome of F_2_ offspring and their parents. Boxplots of the Shannon and Simpson indices, and the number of observed OTUs of fungal (**A**) and bacterial (**B**) communities in the rhizosphere of the cotton plants of F_2_ plants and the four parents

There were significant differences among the two parents and the progeny within crosses for both Shannon and Simpson indices of fungal communities (*P* < 0.001). However, most of the observed variability in both Shannon and Simpson indices was unexplained—86.6% (Shannon index) and 89.9% (Simpson index). Overall, the progeny group for both crosses had higher alpha diversity indices of fungal communities as well as greater variability than the two parents (Fig. 3A). The same trend was also present for the observed number of fungal OTUs per sample (Fig. 3A). For Bray–Curtis beta diversity indices, the parents differed (*P* < 0.001) from F_2_ progeny, accounting for 5.1% of the total variation in the indices. The two crosses also differed (*P* < 0.01) but not between parents of the same cross (*P* = 0.1). Most variability was due to (1) between replicated samples within each parent (9.6%) and (2) between F_2_ progeny plants within each cross (75.4%).

As for fungi, there were significant differences among the two parents and the progeny within crosses for both Shannon and Simpson indices of bacterial communities (*P* < 0.001) although most of the observed variability was not accounted for—89.8% (Shannon index) and 91.7% (Simpson index). Overall, the progeny group for both crosses had higher alpha diversity indices of bacterial communities than the parents (Fig. 3B). The same trend was also present for the observed number of bacterial OTUs per sample (Fig. 3B). Overall, samples from cultivar J11 had smaller indices well as lower variability than the other groups. For beta diversity indices, the parents differed (*P* < 0.001) from F_2_ progeny, accounting for 3.9% of the total variation in the indices. The two crosses also differed (*P* < 0.05) as did the parents of the same cross (*P* < 0.05). As for fungi, most variability was due to (1) between replicated samples within each parent (24.1%) and (2) between F_2_ progeny plants within each cross (68.2%).

### Genetic control of rhizosphere fungal and bacterial microbiome

**Fig. 4:**
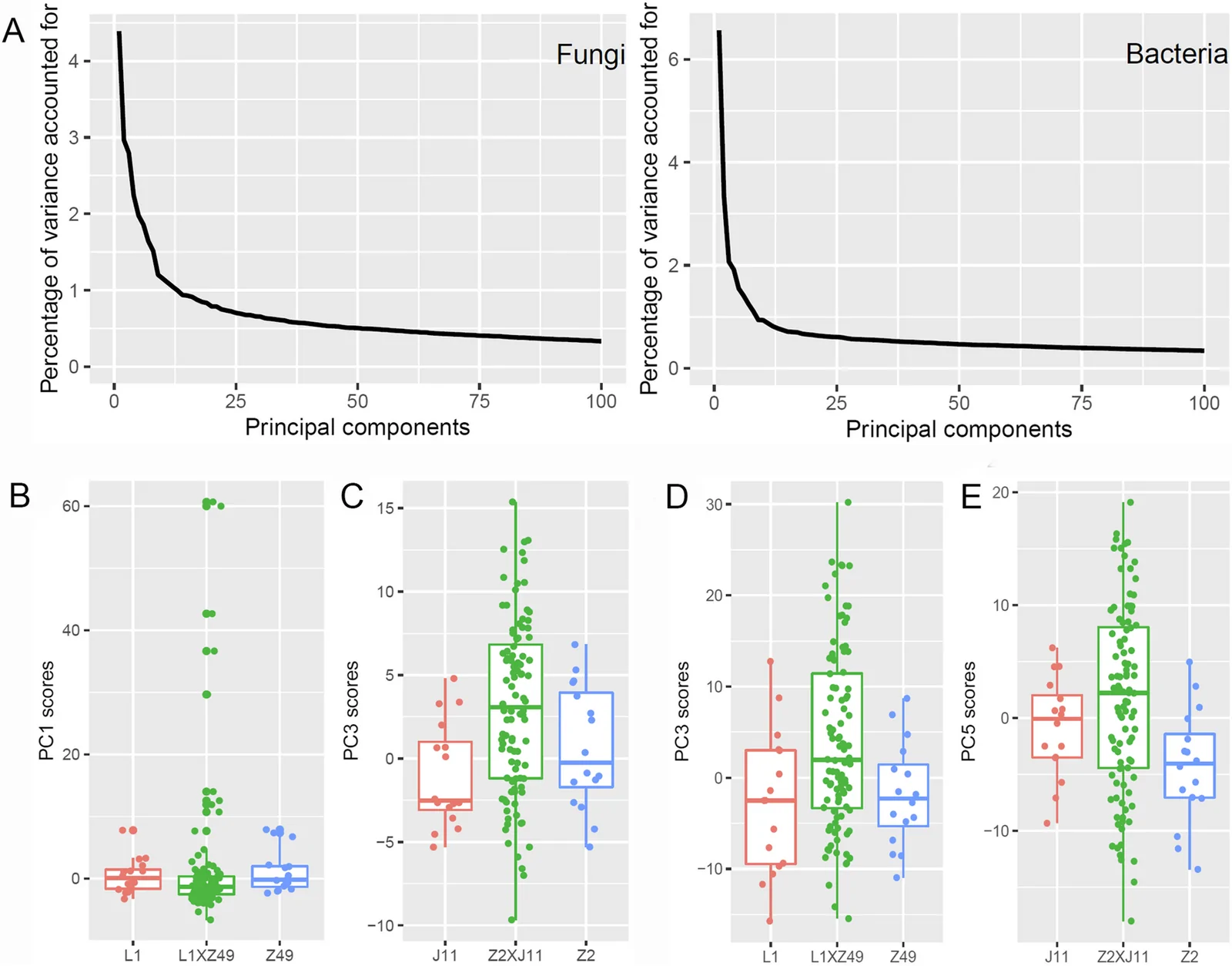
Genetic control of rhizosphere fungal and bacterial microbiome as represented by PC scores. **A** Percentage of variability in the original fungal and bacterial OTU counts data accounted by the first 100 principal components (PCs); logarithm transformed (on the natural base) of normalized (median of ratios) OTU counts data were standardized and then subject to PC analysis (PCA). Boxplots of the fungal PC1 scores for **B** L1 × Z49 and PC3 scores for **C** Z2 × J11 cross where the variance among the 100 progeny samples was much greater than the random variability estimated form the four parents. Boxplots of the bacterial PC3 scores for **D** the L1 × Z49 and PC5 scores for **E** the Z2 × J11 cross where the variance among the 100 progeny samples was much greater than the random variability estimated form the four parents

Of 1487 fungal OTUs, the top 1188 OTUs (accounting for 99.99% of the total number of sequences) were kept and subjected to PCA. Although the first PC only accounted for 4.4% of the total variability, the percentage of variability explained by individual PCs initially declined steeply (Fig. 4A). For L1 × Z49, the variance of the fungal microbiome among the 100 progeny plants (*V*_*T*_) was significantly greater than the random variability (*V*_*E*_) for 12 PCs (Table 1). The two parents did not differ significantly for any of the 12 PCs. Figure 4B gives an example plot for fungal PC1, showing that the progeny displayed a greater variability, particularly towards the high scores. The fungal genetic component as the proportion of the total variability ranged from 0.470 to 0.935. For Z2 × J11, *V*_*T*_ was significantly greater than *V*_*E*_ for 10 fungal PCs (Table 1). Only for two of the 10 PCs did the two parents differ significantly; interestingly for these two PCs, the significance level of *V*_*T*_ > *V*_*E*_ was also the greatest among the 10 fungal PCs. As an example, the progeny not only had a higher average fungal PC3 score than the two parents but also displayed a greater variability (Fig. 4C). The estimated heritability ranged from 0.473 to 0.740. The two crosses shared common 6 fungal PCs for which *V*_*T*_ was significantly greater than *V*_*E*_ (Table 1).

**Table 1:** Summary of analysis of the first 100 principal components (PCs) for fungal community^#^

PC	% variance explained by PC	L1 × Z49		Z2 × J11	
		*P* value: *V*_*T*_ > *V*_*E*_	(*V*_*T*_ − *V*_*E*_)/*V*_*E*_	*P* value: *V*_*T*_ > *V*_*E*_	(*V*_*T*_—*V*_*E*_)/*V*_*E*_
1	4.390	< 0.001	0.935		
2	2.964			0.011	0.538
3	2.796	< 0.001	0.727	< 0.001	0.660
4	2.240	< 0.001	0.810	< 0.001	0.644
17	0.876	< 0.001	0.785		
18	0.848	< 0.001	0.703	< 0.001*	0.686
19	0.834	0.005	0.554	0.041	0.473
22	0.751	< 0.001	0.657	< 0.001*	0.740
25	0.703	0.036	0.475		
26	0.692			0.007	0.559
29	0.659	0.014	0.517		
30	0.655	< 0.001	0.657	0.002	0.599
31	0.634	< 0.001	0.665		
36	0.586			0.022	0.508
51	0.498	0.036	0.470		
61	0.456			0.026	0.496

^#^These 100 PCs of the top 3493 fungal OTUs (accounting for 99.99% of the total number of sequences) for the two crosses, testing whether the variance among 100 progeny samples (*V*_*T*_) is greater than the random variability (*V*_*E*_). The probability value was adjusted for multiple tests (Benjamini-Hochberg)

^*^The two parents also differed significantly

Of 4196 bacterial OTUs, the top 3493 OTUs (accounting for 99.99% of the total number of sequences) were kept and subjected to PCA. Although the first PC only accounted for 6.6% of the total variability, the percentage of variability explained by individual PCs initially declined steeply (Fig. 4A), more than the rhizosphere fungal data. For L1 × Z49, *V*_*T*_ was significantly greater than *V*_*E*_ for 34 bacterial PCs (Table 2) although the two parents did not differ significantly for any of the 34 bacterial PCs. Figure 4D plots bacterial PC3 scores as an example, showing that the progeny displayed a greater variability, particularly towards the high scores, as well as a higher average. The broad sense heritability ranged from 0.408 to 0.933. For Z2 × J11, *V*_*T*_ was significantly greater than *V*_*E*_ for 20 PCs (Table 2), but the two parents did not differ significantly for any of the 20 PCs. Figure 4E uses bacterial PC5 as an example to illustrate that the progeny not only had a higher average score than the two parents but also displayed greater variability. The broad sense heritability ranged from 0.477 to 0.784. The two crosses shared common bacterial 16 PCs for which *V*_*T*_ was significantly greater than *V*_*E*_ (Table 2).

**Table 2:** Summary of analysis of the first 100 principal components (PCs) for bacterial community^#^

PC	% variance explained by PC	L1 × Z49		Z2 × J11	
		*P* value: *V*_*T*_ > *V*_*E*_	(*V*_*T*_ − *V*_*E*_)/*V*_*E*_	*P* value: *V*_*T*_ > *V*_*E*_	(*V*_*T*_ − *V*_*E*_)/*V*_*E*_
2	3.332	0.006	0.510		
3	2.068	0.001	0.586		
4	1.915	0.005	0.520	0.015	0.490
5	1.549	0.002	0.561	< 0.001	0.664
8				0.012	0.505
9				0.019	0.477
10	0.933	< 0.001	0.774	0.001	0.616
11	0.872	< 0.001	0.684	0.006	0.536
12	0.813	< 0.001	0.773	0.001	0.600
13	0.773	< 0.001	0.933	0.018	0.480
14	0.741	< 0.001	0.779		
15	0.710	< 0.001	0.852	0.001	0.614
16	0.704	< 0.001	0.833	0.001	0.611
17	0.696	< 0.001	0.810		
18	0.669	< 0.001	0.627	< 0.001	0.679
19	0.657	0.017	0.465		
20	0.648	0.044	0.408	< 0.001	0.784
21	0.634	0.042	0.412	< 0.001	0.647
22	0.626	< 0.001	0.824		
23	0.616	< 0.001	0.832	< 0.001	0.703
24	0.610	< 0.001	0.831	0.013	0.500
25	0.607	< 0.001	0.691		
26	0.600	< 0.001	0.644		
28	0.568	< 0.001	0.741	< 0.001	0.716
29	0.563	< 0.001	0.624	< 0.001	0.684
30	0.560	< 0.001	0.694		
31				0.011	0.513
32	0.551	0.002	0.561		
33	0.549	0.004	0.528		
34	0.542	< 0.001	0.621		
35	0.537	0.032	0.428		
37	0.523	0.002	0.545		
38	0.517	0.002	0.546		
41	0.507	0.001	0.574		
42				0.013	0.498
43	0.497	< 0.001	0.607	0.004	0.558
48	0.476	0.029	0.436		
96	0.349	0.023	0.448		

^#^These 100 PCs of the top 3493 bacterial OTUs (accounting for 99.99% of the total number of sequences) for the two crosses, testing whether the variance among 100 progeny samples (*V*_*T*_) is greater than the random variability (*V*_*E*_). The probability value was adjusted for multiple tests (Benjamini-Hochberg)

### Genetic control of top 100 fungal OTUs and bacterial OTUs

For L1 × Z49, *V*_*T*_ was significantly greater than *V*_*E*_ for 29 out of the top 100 fungal OTUs (Table 3), five of which cannot be assigned to the phylum level. The two parents did not differ significantly for any of these OTUs. The broad sense heritability ranged from 0.414 to 0.788. For Z2 × J11, *V*_*T*_ was significantly greater than *V*_*E*_ for 24 out of the top 100 fungal OTUs (Table 3), three of which cannot be assigned to the phylum level. The two parents did not differ significantly for any of these OTUs. The broad sense heritability ranged from 0.424 to 0.841. For 14 fungal OTUs, *V*_*T*_ was significantly greater than *V*_*E*_ for both crosses, including *Trichoderma brevicompactum* and* V*. *dahliae* (Table 3).

**Table 3:** Summary of analysis of the top fungal 100 fungal OTUs^#^

OTU_ID	Taxonomy*	L1 × Z49		Z2 × J11	
		*P* value: *V*_*T*_ > *V*_*E*_	(*V*_*T*_ − *V*_*E*_)/*V*_*E*_	*P* value: *V*_*T*_ > *V*_*E*_	(*V*_*T*_ − *V*_*E*_)/*V*_*E*_
OTU106	*Fusarium* (g)	0.022	0.477	0.022	0.472
OTU12	*Verticillium dahliae*	0.005	0.550	0.046	0.424
OTU127	*Trichoderma* (g)	0.015	0.502		
OTU1439	*Fusarium* (g)	0.046	0.414	0.004	0.559
OTU1481	*Cryptococcus* (g)	0.035	0.430		
OTU1595	*Microascaceae* (f)	< 0.001	0.698	< 0.001	0.809
OTU21	*Pseudogymnoascus pannorum*	0.022	0.480		
OTU239	*Fungi* (k)	0.035	0.431		
OTU24	*Phaeosphaeria fuckelii*	< 0.001	0.668		
OTU27	*Trichoderma brevicompactum*	0.026	0.463	0.034	0.445
OTU304	*Fungi* (k)	0.001	0.621		
OTU32	*Dothideomycetes* (c)	0.031	0.449	0.007	0.527
OTU430	*Mortierellales* (o)	< 0.001	0.717		
OTU445	*Fungi* (k)	0.032	0.442	< 0.001	0.701
OTU452	*Alternaria alternata*	0.031	0.449	0.007	0.532
OTU51	*Corynespora cassiicola*	< 0.001	0.684	0.022	0.474
OTU53	*Fungi* (k)	0.022	0.475	0.002	0.611
OTU55	*Fungi* (k)	< 0.001	0.788	0.003	0.57
OTU6	*Fusarium* (g)	0.035	0.432		
OTU60	*Mortierella alpina*	0.031	0.452		
OTU65	*Neonectria* (g)	0.001	0.607	0.005	0.546
OTU727	*Cladosporium cladosporioides*	0.010	0.522		
OTU75	*Mortierella alpina*	0.020	0.486		
OTU8	*Fusarium solani*	0.003	0.568		
OTU81	*Truncatella angustata*	0.034	0.438		
OTU82	*Gymnoascus reesii*	0.031	0.446		
OTU83	*Rhizoctonia solani*	0.011	0.516	0.025	0.464
OTU88	*Acremonium* (g)	0.004	0.562	< 0.001	0.841
OTU91	*Ascomycota* (p)	0.002	0.593		
OTU119	*Hypocreales* (o)			0.043	0.430
OTU1201	*Ascomycota* (p)			0.027	0.457
OTU1519	*Metarhizium anisopliae*			0.003	0.583
OTU22	*Hannaella luteola*			0.010	0.512
OTU28	*Cercospora coniogrammes*			0.003	0.570
OTU34	*Dothideomycetes* (c)			0.003	0.570
OTU37	*Hannaella sinensis*			0.009	0.516
OTU587	*Chaetomium* (g)			0.003	0.577
OTU61	*Acremonium acutatum*			0.002	0.604
OTU9	*Ascomycota* (p)			0.016	0.490

^#^Summary of analysis of the top fungal 100 OTUs (with the highest counts), testing whether the variance among 100 progeny samples (VT) is greater than the random variability (VE). The probability value was adjusted for multiple tests (Benjamini-Hochberg)

^*^k kingdom, *p* phylum, *o* order, *c* class, *f* family, *g* genus

For L1 × Z49, *V*_*T*_ was significantly greater than *V*_*E*_ for 10 out of the top 100 bacterial OTUs (Table 4). Broad sense heritability ranged from 0.475 to 0.728. For Z2 × J11, *V*_*T*_ was significantly greater than *V*_*E*_ for only one OTU (*Achromobacter mucicolens*) with its broad sense heritability being 0.615.

**Table 4:** Summary of analysis of the top 100 bacterial OTUs^#^

OTU_ID	Taxonomy*	L1 × Z49		Z2 × J11	
		*P* value: *V*_*T*_ > *V*_*E*_	(*V*_*T*_ − *V*_*E*_)/*V*_*E*_	*P* value: *V*_*T*_ > *V*_*E*_	(*V*_*T*_ − *V*_*E*_)/*V*_*E*_
OTU1396	*Rhizobiales* (o)	0.000	0.646		
OTU187	*Povalibacter uvarum*	0.007	0.554		
OTU24	*Luteimonas cucumeris*	0.031	0.489		
OTU2479	*Streptomyces* (g)	0.000	0.728		
OTU26	*Ilumatobacter* (g)	0.000	0.671		
OTU51	*Myxococcales* (o)	0.017	0.518		
OTU54	*Steroidobacter denitrificans*	0.000	0.698		
OTU59	*Actinomycetales* (o)	0.004	0.576		
OTU88	*Catellatospora* *methionotrophica*	0.000	0.653		
OTU887	*Bacillus niacini*	0.039	0.475		
OTU57	*Achromobacter mucicolens*			0.005	0.615

^#^Summary of analysis of the top bacterial 100 OTUs (with the highest counts), testing whether the variance among 100 progeny samples (*V*_*T*_) is greater than the random variability (*V*_*E*_). The probability value was adjusted for multiple tests (Benjamini-Hochberg)

^*^k kingdom, *p* phylum, *o* order, *c* class, *f* family, *g* genus

## Discussion

The dominant bacterial phylum in the cotton rhizosphere was *Proteobacteria*, accounting for 64.4% of the sequence reads, which is consistent with previous findings in the cotton rhizosphere (Wei et al. 2021, 2019). *Proteobacteria* are generally adapted to the plant rhizosphere and across different plant species (Peiffer et al. 2013) and respond to labile carbon sources, and are usually considered to be r-selected or rapidly growing microbiota (Fierer et al. 2007). With regard to rhizosphere fungi, nearly 53% of the fungal sequences could not be assigned to the phylum level at the 90% confidence level; most of the other sequences were assigned to *Ascomycota* (25.5%) or *Basidiomycota* (20.9%), similar to the strawberry rhizosphere (Wei et al. 2016).

### Microbial diversity in rhizosphere for parental and F_2_ offspring cotton plants

For rhizosphere bacteria and fungi, we demonstrated that average alpha diversity indices for F_2_ offspring progeny for both crosses were higher than for the two parents; furthermore the variability among samples in the alpha diversity indices was also greater for progeny samples than for parents. A previous study reported that fungal communities in the rhizospheres of hybrid maize plants had higher alpha diversity than inbred lines but no consistent differences in alpha diversity of rhizosphere bacteria between inbred lines and hybrids (Wagner et al. 2020). In addition, we found significant genetic components for specific rhizosphere microbiome components of cotton, often with moderate to high broad sense heritability (> 0.4), but these components were only a minor proportion of the entire microbiome. This can be seen from the small number of PCs or OTUs with significant genetic components. These results indicate that specific components of the cotton rhizosphere microbiome are genetically controlled by polygenic properties; even when microbes could be reliably transmitted to the F_2_, most of them disappeared. Similar findings indicate significant differences in the maize inbred lines, but the heritability level was low and the genetic relationship among the inbred lines was not correlated with the diversity characteristics of the rhizosphere microbiome (Peiffer et al. 2013). The overall low heritability of the phytobiome may be due to strong environmental effects (Clouse and Wagner 2021). Meanwhile, microbe-microbe interactions have a strong influence on plant–microbe interactions (Kroll et al. 2017), complicating their predictability. Plants are subjected to a variety of biotic and abiotic stresses, which can induce changes in transcriptomics and metabonomics, leading to changes in root and leaf exudates, thereby influencing phytobiomes (Liu et al. 2020). Greenhouse or controlled environment growth chambers may be able to regulate climate fluctuations and reduce soil heterogeneity than under field conditions and thus could lead to greater heritability estimates.

### Specific rhizosphere components are likely to be genetically controlled by plants

In the present study, transgressive segregation was common for those PCs or OTUs with significant genetic component, indicating a polygenic nature of the genetic control. Peiffer et al. (2013) speculated that the maize microbial community is controlled by several major genes, rather than many minor genes in the whole genome, which is used to explain the lack of correlation of maize genetic relationships with microbial diversity. Studies with *Arabidopsis* mutants and transgenes have shown that leaf cuticle characteristics affected microbial colonization of microbes (Ritpitakphong et al. 2016) and that physiological traits such as hormone signaling and defensive secondary chemistry affected the rhizosphere microbiome (Lebeis et al. 2015). Further research is needed to assess both plant traits and phytobiome characteristics to determine their relationships, which may shed lights on the nature of the control of the phytobiome by plants as observed in the present study.

The main purpose of the present study was to determine the extent of host genetic control of the rhizosphere microbiome through analysis of principal components and most abundant OTUs. Analysis of PCA scores represents directly the most variable components of the rhizosphere microbiome whereas most abundant OTUs represent common individual microbial groups in the rhizosphere. Thus, genetic analysis of these two types of data should be able to reveal the extent of host genetic control of rhizosphere microbiome. Analysis of individual less-abundant OTUs (already included in PCAs) in genetic analysis will not change the conclusions from PCAs and most abundant OTUs. In addition, inherently it becomes gradually more difficult to detect significant differences among samples when relative abundance of individual OTUs reduces. We showed that the overall plant genetic component was stronger for cotton rhizosphere fungi than for rhizosphere bacteria, which could be related to the limited capacity of dispersion of bacterial communities as compared to fungal communities with their hyphal growth and branching. For many microbial groups, albeit still a small proportion of the entire microbiome, the variability among F_2_ offspring is greater than random variability, indicating that specific rhizosphere microbial groups are likely to be genetically controlled by plants. Some of the taxa with significant genetic control by plants have been linked to positive or negative effects on plant health. These microbes include *V. dahliae*, *Rhizoctonia solani*, *Alternaria alternata* (Singh et al. 2020), and *Fusarium*, which are fungal pathogens of cotton, but also include well-known biocontrol agents of soilborne diseases, such as *Trichoderma brevicompactum* (Shentu et al. 2014), *Streptomyces* (Niu et al. 2016), and *Rhizobiales* (Erlacher et al. 2015). Thus, plant genotypes may affect the relative abundance of pathogens and beneficial microbes, influencing plant susceptibility and disease development.

There was a significant genetic component in a number of PCs, which were determined by many microbes. This was most likely due to two reasons. Firstly, plant traits that are expected to affect microbiome composition and activity, such as root exudates (Zhalnina et al. 2018) and root architecture (Saleem et al. 2018), are complex in nature and may be controlled by many genes. Specific root exudates would selectively attract those microbes that could directly or indirectly use the exudates metabolically (Huang et al. 2014). Root exudates and other root deposits secreted by host plants influence rhizosphere composition (Moe 2013). Not all root exudates are directly involved in plant nutrition and growth. Some of them act as signaling molecules to mediate interactions in root communities (Olanrewaju et al. 2019). Secondly, many microbes may share similar environmental requirement for their development.

Although much progress in manipulating crop microbiomes has been made recently, further research is still needed to implement holobiont-level breeding effectively. Present results suggested that exploiting rhizosphere microbiome for sustainable agriculture via breeding could be difficult. This can be summarized in one phrase: a one-to-many relationship. One selection criterion may select for or against many microbes (e.g., those contributing significantly to specific microbiome features as captured by PCs). These affected microbes may include beneficial and pathogenic microbes, as demonstrated by the present study. Moreover, many microbes that may be co-selected but with unknown identities and functions. Therefore, in the short term, soil amendment with specific microbial consortia may be the way forward to improve soil conditions and support intensive agriculture. It should be noted that understanding how plant genetic variation interacts with such potential augmentative biological agents will still be an important concern. This is similar to augmented application of biocontrol microbes to manage pathogens or inundative release of predator to control pests. To improve the persistence of these introduced beneficial microbes, research is needed to understand their ecological requirement and how they interact with resident phytobiome.

In this work, we characterized the rhizosphere composition of cotton F_2_ offspring plants of two crosses and demonstrated that specific rhizosphere components are likely to be genetically controlled by plants when the rhizosphere microbiome was characterized as PCs or individual top microbial groups were considered. More encouragingly, there are common microbiome components (i.e., PCs) and specific microbial groups with a significant genetic component between the two crosses.

## Supplementary Information

Below is the link to the electronic supplementary material.

Supplementary file1 (PDF 184 KB)

## Data Availability

The raw sequencing data reported in this paper are publicly available in the NCBI Sequence Read Archive (SRA) under the BioProject number PRJNA756865.
