# Supplementary material for: Genetic control of rhizosphere microbiome of the cotton plants under field conditions
Source: Appl Microbiol Biotechnol. 2024 Jun 11;108(1):371. doi: 10.1007/s00253-024-13143-0 (PMC11166756; doi:10.1007/s00253-024-13143-0)
Supplement: Supplementary file 1 — Supplementary file1 (PDF 184 KB) [file 253_2024_13143_MOESM1_ESM.pdf]

Name of journal: Applied Microbiology and Biotechnology

Genetic control of rhizosphere microbiome of the cotton plants under field conditions

Feng Wei [1,2,3] • Zili Feng [1,2] • Chuanzhen Yang [1] • Lihong Zhao [1] • Yalin Zhang [1,2] • Jinglong Zhou [1] • Hongjie Feng [1,2,3 *] • Heqin Zhu [1,2,3 *] • Xiangming Xu [4]

[1] National Key Laboratory of Cotton Bio-breeding and Integrated Utilization, Institute of Cotton Research of Chinese Academy of Agricultural Sciences, Anyang 455000, China

[2] Western Agricultural Research Center, Chinese Academy of Agricultural Sciences, Changji, 831100, Xinjiang, China

[3] School of Agricultural Sciences, Zhengzhou University, Zhengzhou 450001, China

[4] NIAB East Malling Research, Kent ME19 6BJ, UK

[*] **Correspondence author:** Heqin Zhu, Email: heqinanyang@163.com, Tel: 86 0372 2562280; Hongjie Feng, Email: fenghongjie@caas.cn, Tel: 86 0372 2562235.

Supplementary material

Table S1. Wilt scores of individual plants in field.

Plant materials	Number of plants with different disease scores					Total number of plants
	0	1	2	3	4	
cv. Z2	68	19	11	2	0	100
cv.J11	8	8	22	51	11	100
Z2×J11	23	27	28	17	5	100
cv. L1	15	13	25	39	8	100
cv. Z49	52	29	15	4	0	100
L1×Z49	31	34	19	14	2	100

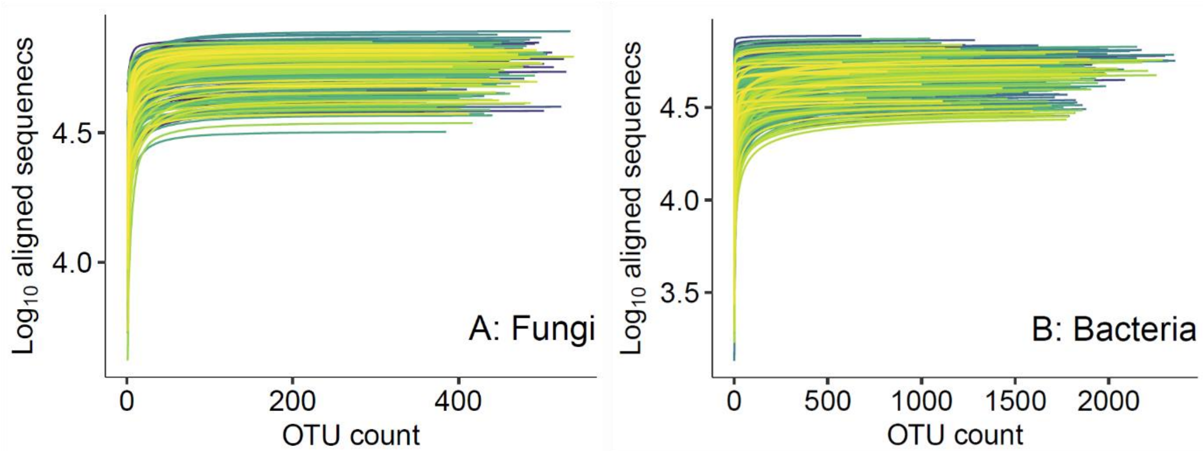

Fig. S1. Rarefaction curves for the full study. Rarefication curves showing the accumulated sequence reads against the number of fungal (A) and bacterial (B) OTUs, which indicates that sequencing depth is sufficient for all samples.
